# Autophagy and mitochondrial dysfunction in adjuvant-arthritis rats treatment with resveratrol

**DOI:** 10.1038/srep32928

**Published:** 2016-09-09

**Authors:** Junqiang Zhang, Xianbin Song, Wei Cao, Jinseng Lu, Xiaoqing Wang, Gaoyuan Wang, Zhicheng Wang, Xiaoyu Chen

**Affiliations:** 1 Department of Histology and Embryology, Anhui Medical University, Hefei 230032, China; 2 Basic Medicine, Anhui Medical College, Hefei 230601, China; 3 Department of Orthopaedic, the First Affiliated Hospital of Anhui Medical University, Hefei 230031, China; 4 Department of Laboratory Medicine, Huashan Hospital, Shanghai Medical College, Fudan University, Shanghai 200040, China

## Abstract

Resveratrol is a polyphenol derivatives which exhibits a pro-apoptotic effect in a variety of human cancers by triggering mitochondria apoptosis pathway and autophagy. However, there are scarcely reports on its apoptosis-promoting effect in abnormal proliferation fibroblast-like synoviocytes (FLSs). In this study, we investigated the underlying mechanism and apoptosis-inducing effects of resveratrol on the abnormal proliferation of FLSs in adjuvant-arthritis (AA) rats. Since using resveratrol for 12 days resulted in a significant decreasing the swelling degree of the paw, reducing malondialdehyde (MDA) content and enhancing superoxide dismutase (SOD) activity, antioxidant capacity, glutathione peroxidase and glutathione reductase ratio in AA rats. Moreover, we found that 5 μMH_2_O_2_ could increase cells viability, Beclin1, LC3A/B, MnSOD, SIRT3 protein expression in FLSs. But, resveratrol could reverse these effects by changing mitochondrial membrane potential (Δψm) to promote mitochondrial reactive oxygen species (mtROS) generation in 5 μMH_2_O_2_-treatment FLSs. These results suggest that oxidative stress existed in AA rats. Resveratrol could suppress oxidative stress in AA rats and increase mtROS production by reducing autophagy protein Beclin1, LC3A/B and oxidative stress protein MnSOD to promoted the apoptosis of FLSs. Thus, targeting of mtROS may be a crucial mechanism of resveratrol confers patients with rheumatoid arthritis.

Rheumatoid arthritis (RA) is a chronic systemic autoimmune disease characterized by persistent fibroblast-like synoviocytes (FLSs) proliferation with inflammatory cell infiltration and joint destruction^1^. Currently, adjuvant-induced arthritis (AA) rats are the most widely used arthritis models in academia and industry^1^. AA, which consist of paraffin oils, mannide monooleate, and heat-killed mycobacteria (Mb), known as complete Freund’s adjuvant (CFA), is an experimental model of rheumatoid arthritis^2^. Some evidences show that FLSs exhibits tumor like growth, which is manifested as lack of contact inhibition^3^. Inflammatory cytokines such as tumor necrosis factor alpha (TNF-α), interleukin-6 (IL-6) and interleukin-1 beta (IL-1β) were significantly increased in patients with RA^4^. Some evidences indicated that over production of proinflammatory cytokines stimulated neutrophils and activated macrophages secreting ROS to the synovial fluid, which may act as mediators of synovial hyperplasia^5^,^6^. So promoting the apoptosis of FLSs, which may transform tissue composition and homeostasis, affecting the pathogenesis of RA.

Reactive oxygen species (ROS) such as superoxide anion (O_2_•^−^), and hydroxyl radical (OH•), Hydroperoxyl (HO_2_•), Peroxyl (ROO•), Alkoxyl (RO•), hydrogen peroxide (H_2_O_2_), plays a role in various biological processes including proliferation, Oxidative Stress, autophagy, apoptosis, tumor development^7^,^8^,^9^. Intracellular 90% ROS was produced by mitochondria in physiological conditions^10^. That Prolonged exposed in low levels of ROS may induce somatic mutations and cancer progression could contribute to acquire a malignant phenotype^11^. But, high levels of ROS lead to DNA and protein damage, lipid peroxidation, apoptosis even necrosis^12^. The enzymatic antioxidant defences mainly include Superoxide Dismutase (SOD), Glutathione Peroxidase (GSH-PX) and glutathione reductase (GR) in the body^12^. Superoxide or superoxide anion can be dismutated to H_2_O_2_ in a reaction catalyzed by superoxide dismutase (SOD)^13^. GSH-PX can promote the formation of glutathione (GSH) and H_2_O_2_ reaction to produce H_2_O and glutathione disulfide (GSSG). Under the catalysis of glutathione reductase (GR), the glutathione disulfide generates glutathione with H^+^ which was provided by NADPH^12^. Furthermore, some studies reported that alterations in extracellular GSH/GSSG could affect proliferation of colorectal carcinoma and lung fibroblast cells^14^,^15^. So the activity of GSH and GR are very important. Due to the imbalance between oxidation system and antioxidation system increased chemical reaction or insufficient antioxidant defence system results in oxidative stress^16^. Some studies suggested mitochondrial ROS (mtROS) directly regulated the composition of autophagosomes^17^. Although the regulation of autophagy is unknown, it is highly likely that it is affected by oxidative modification of transcription factors^17^. Excessive generation of ROS cause mitochondrial damage resulting in the loss of its function and then cause mitochondrial autophagy. Mitochondrial autophagy remove damaged mitochondria by preventing mtROS accumulating which is a scavenger to maintain normal mitochondrial function^18^.

Resveratrol (3,5,4′-trihydroxy-trans-stilbene, Res) is a natural polyphenol abundantly found in grape skins and polygonum cuspidatum which possesses a variety of biochemical and physiological effects including anti-inflammatory, anti-oxidation, anti-proliferation and chemopreventive^18^. Low concentration of resveratrol is an excellent scavenger of hydroxyl, superoxide, and other radicals. Resveratrol is also able to avoid excessive ROS induced lipid peroxidation and DNA damage^19^. It is well known that a high dose of resveratrol can induce ovarian cancer cells apoptosis^20^,^21^. Silent mating-type information regulation 2 homolog 3 (Sirt3) is the primary mitochondrial deacetylase^22^ and directly regulates biological functions involved in mitochondrial energy production^23^ which also plays a key role in regulating mtROS homeostasis. Some seminal papers have been reported that Sirt3 regulates manganese superoxide dismutase (MnSOD) deacetylation and identified the target lysines^24^,^25^,^26^. At the same time, lysine acetylation is also involved in the regulation of p53 which also plays a role in mitochondrial redox regulation^27^. The study also showed that the forms of oxidative stress induce Sirt3 deacetylation activity and MnSOD activity^28^. It is well established that over expression of Sirt3 decreases both intracellular mitochondrial O_2_•^−^ and total ROS levels, suggesting that Sirt3 regulates both energy production and mtROS scavenging pathways^29^. Some research pointed that mitochondrial oxidative stress induced compensatory upregulation of SIRT3, which might in turn activate phosphorylation of AMPK and subsequently triggered autophagy by upregulating Beclin1 expression and LC3 II/I conversion^30^. Furthermore, Res induced apoptosis via ROS-triggered autophagy in human colon cancer cells^31^, while the study indicated Res enhanced temozolomide-induced apoptotic cell death in malignant glioma by inhibiting autophagy^32^. Similarly, It is unknown whether resveratrol can regulates mtROS level to promote FLSs apoptosis by Sirt3-MnSOD axis or autophagy pathway in H_2_O_2_-treatment FLSs.

## Results

### Arthritis induction in SD rats

**Figure 1:**
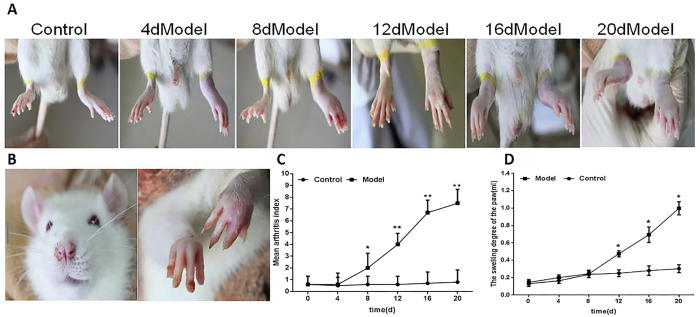
Arthritis induction in SD rats. (**A**) Representative images show the effect on paw edema in rats with FCA-induced arthritis. (**B**) the nose and the double fore legs inflammation secondary injury in FCA-induced SD rats. (**C**) The severity of FCA-induced arthritis in SD rats. (**D**) The swelling degree of the paw in SD rats with FCA-induced arthritis, as determined by toe swelling apparatus calculated at the indicated time points. Values are the means ± SD of at least three independent experiments. *P < 0.05; **P < 0.01 versus control. n = 15/group.

The Fig. 1 displays that after the injection FCA in SD rats 20 days, compared with the secondary began to appear obvious swollen (Fig. 1A) and the nose and double fore legs appear different degree of inflammation injury (Fig. 1B). Besides, after the injection for 8, 12, 18, 20 days, compared with the control group, arthritis index was gradually increased (Fig. 1C) and the swelling degree of the paw was gradually increased, too (Fig. 1D).

### Resveratrol reduces oxidative injury parameters in AA rats

**Figure 2:**
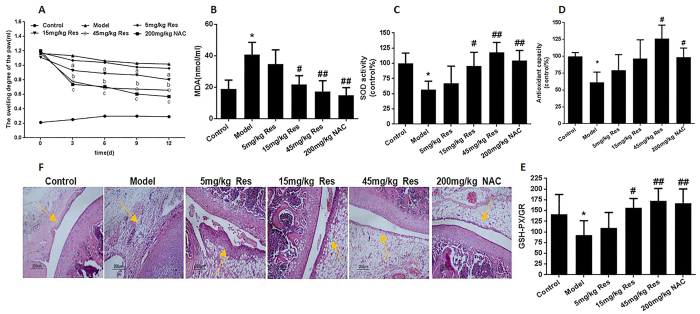
Resveratrol reduces oxidative injury in AA rats. after injecting FCA 20 days, treated with 5 mg/kg, 15 mg/kg, 45 mg/kg resveratrol and 200 mg/kg N-acetyl-L-cysteine (NAC) for 12 days by continuous intragastric administration. (**A**) The swelling degree of the paw in SD rats after intragastric administration. (**B**) Lipoperoxide levels in the serum from six groups rats. (**C**) SOD activity in six groups rats serum (**D**) Antioxidant capacity in six groups rats serum (**E**) The ratio of glutathione peroxidase and glutathione reductase in six groups rats serum. (**F**) HE staining of knee joint in AA rats after administration resveratrol. Data represent the means, ^a^P < 0.05, ^b^P < 0.01, ^c^P < 0.01 versus model. Values are the means ± SD of at least three independent experiments. *P < 0.05 versus control; ^#^P < 0.05, ^##^P < 0.01 versus model. n = 10/group.

After successively intragastric administration of 5 mg/kg, 15 mg/kg and 45 mg/kg resveratrol and acetyl-l-cysteine (NAC, 200 mg/kg) for 12 days. Compared with the model group, 5 mg/kg resveratrol group of toes swelling didn’t change significantly (P > 0.05). But, the change of 15 mg/kg resveratrol group was significantly decreased (P < 0.05), and 45 mg/kg resveratrol and 200 mg/kg NAC group changes were most obvious (p < 0.01), after 12 days can reach 0.80 ml, 0.65 ml, 0.41 ml (Fig. 2A). And the content of MDA in the model group was about 2 folds more than control group. After administration 5 mg/kg, 15 mg/kg, 45 mg/kg resveratrol and 200 mg/kg NAC the contents of MDA were gradually decreased in rats serum (Fig. 2B). And the control of total antioxidant capacity (T-AOC) (Fig. 2C), total SOD activity (T-SOD) (Fig. 2D), the ratio of glutathione peroxidase and glutathione reductase (GSH-PX/GR) (Fig. 2E) was significantly higher than the model group. Compared with the model group, 15 mg/kg, 45 mg/kg resveratrol and 200 mg/kg NAC group was significantly increased. In model group, the synovial tissue obviously thickened, and the cartilage tissue was eroded. A large number of fragments appeared in the synovial cavity. However, after treatment with resveratrol, the above symptoms relieved or even disappeared (Fig. 2F).

### Resveratrol inhibited H_2_O_2_-treatment FLSs proliferation

**Figure 3:**
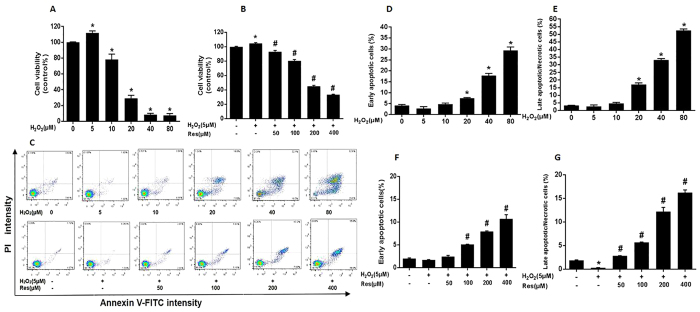
Resveratrol inhibited H_2_O_2_-treatment FLSs proliferation. (**A,B**) Cells were treated with different concentrations (0, 5, 10,20, 40 and 80 μM) H_2_O_2_ for 12 h and cells were pretreated for 24 h with various concentrations of Res (0, 50,100,200 and 400 μM) before treatment with 5 μMH_2_O_2_ incubation 12 h, respectively. Cell viability was determined using the CCK-8 assay and data are expressed as percentage of the control. (**C**) Representative images of flow cytometric analysis by Annexin V-FITC/PI staining. The bottom right quadrant represents Annexin V-FITC-stained cells (early-phase apoptotic cells) and the top right quadrant represents PI- and FITC-dual-stained cells (late-phase apoptotic or necrotic cells). (**D–G**) Quantitative determination of early apoptotic cell death as the number of Annexin V-FITC-positive cells with PI-negative cells, and late apoptotic/necrotic cell death as the number of Annexin V-FITC-positive and PI-positive cells. Values are the means ± SD of at least three independent experiments. ^*^P < 0.05 versus control; ^#^P < 0.05 versus 5 μMH_2_O_2_ group.

High dosage of H_2_O_2_ can effectively reduce the activation of FLSs. With different concentrations of H_2_O_2_ treatment for 12 h, we set the control group cells as 100%, 5 μMH_2_O_2_ group increased cell activity to 111%, 10–20 μM H_2_O_2_ can reduce the viability to 78%, 29%. 40–80 μMH_2_O_2_ of cell viability was only 8% (Fig. 3A). Before adding 5 μMH_2_O_2_ treatment for 12 h, different concentrations of resveratrol pretreatment for 24 h, 50–400 μMRes can inhibite the proliferation of FLSs. 5 μMH_2_O_2_ cell activity enhanced to 105.1%, after adding resveratrol to cells, the viability reduced to 93.2%, 80.6%, 45.2%, 32.3%, in a dose-dependent (Fig. 3B). The same, 0–10 μMH_2_O_2_ early apoptotic cells and late apoptotic/necrotic cells with few changes. Compared with the control group, 20–80 μM H_2_O_2_ markedly increase early apoptotic cells and late apoptotic/necrotic cells (Fig. 3C–E). Compared with the 5 μMH_2_O_2_ treatment group, Res also can increase 5 μMH_2_O_2_ treatment-FLSs early apoptotic cells and late apoptotic/necrotic cells (Fig. 3C,F,G).

### Resveratrol enhanced mitochondrial superoxide generation and the loss of mitochondrial membrane potential in FLSs

**Figure 4:**
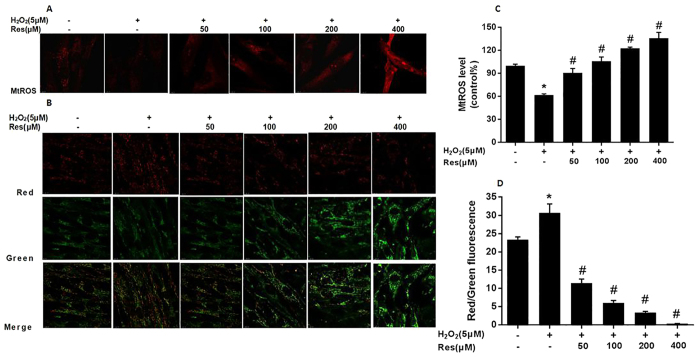
Resveratrol enhanced mitochondrial superoxide generation and the loss of mitochondrial membrane potential in FLSs. Cells were treated with different concentrations (0, 5, 10, 20, 40 and 80 μM) H_2_O_2_ for 12 h and cells were pretreated for 24 h with various concentrations of Res (0, 50, 100, 200 and 400 μM) before treatment with 5 μMH_2_O_2_ incubation 12 h, respectively. (**A**) The mitochondrial superoxide levels were detected using MitoSOX Red and the fluorescence images was carried out using confocal laser scanning microscopy. (**B**) Δψm was determined using confocal laser scanning microscopy. Red fluorescence represents JC-1 aggregates in normal mitochondria, whereas green fluorescence represents JC-1 monomers, indicating unhealthy mitochondria. Merged images indicate JC-1 aggregates and monomers. (**C**) The histogram show quantification of the mitochondrial superoxide levels expressed as the percentage change relative to the control group. (**D**) Δψm in each group was estimated as the red/green fluorescence. Values are the means ± SD of at least three independent experiments. *P < 0.05 versus control; ^#^P < 0.05 versus 5 μMH_2_O_2_ group.

The effects of resveratrol on regulation of mitochondrial redox status and mitochondrial membrane potential (Δψm) were evaluated using MitoSOX Red (Invitrogen, USA) and assay kit with JC-1 (Life technologies, USA) in FLSs. Once MitoSOX Red reagent is selectively targeted to mitochondriain, MitoSOX Red is oxidized by O_2_•^−^ and exhibits red fluorescence. The 5 μMH_2_O_2_ group significantly decreased mitochondrial O_2_•^−^ generation compared with the control group, which was notably increased by resveratrol pretreatment (Fig. 4A,C). JC-1 can selectively enter mitochondria and reversibly changes color as the Δψm changes. There was a significantly increased red fluorescence in 5 μMH_2_O_2_ group. However, Res pretreatment promoted green fluorescence generation, suggesting that the mitochondrial membrane was depolarized (Fig. 4B,D).These results suggested that resveratrol remarkably enhanced collapse of mitochondrial membrane potential in FLSs (P < 0.05).

### Resveratrol declined autophagy related Beclin1 and LC3A/B proteins expression

**Figure 5:**
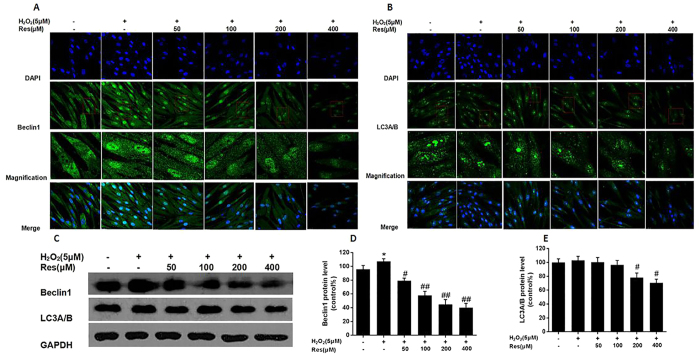
Resveratrol declined autophagy related Beclin1 and LC3A/B proteins expression. Cells were treated with different concentrations (0, 5, 10, 20, 40 and 80 μM) H_2_O_2_ for 12 h and cells were pretreated for 24 h with various concentrations of res (0, 50, 100, 200 and 400 μM) before treatment with 5 μMH_2_O_2_ incubation 12 h, respectively. (**A,B**) Representative images show Beclin1 and LC3A/B proteins localization in FLSs by immunofluorescence assay. Green fluorescence indicates Beclin1 and LC3A/B. Blue fluorescence indicates nucleistained with DAPI. (**C**) After the indicated treatments, the protein was harvested to detect Beclin1 and LC3A/B levels by western blot analysis. (**D,E**) Beclin1/GADPH and LC3A/B/GAPDH. Values are the means ± SD of at least three independent experiments. *P < 0.05 versus control;^#^P < 0.05 versus 5 μMH_2_O_2_ group.

Immunofluorescence images indicated that the control group Beclin1 protein was located in the cytoplasm and abundantly existed in the nucleus surrounding. LC3A/B protein mainly enriched in nucleus and few part presented in the cytoplasm. However, the 5 μMH_2_O_2_ group Beclin1 protein Mainly enriched in cytoplasm and nucleus, especially the Beclin1 fluorescence is very strong in the nucleus and with increasing concentration of Res, Beclin1 and LC3A/B fluorescence intensity gradually reduced (Fig. 5A,B). Western blot analysis, compared with control group, 5 μMH_2_O_2_ can increase the expression of Beclin1 and LC3A/B protein. With increasing concentration of resveratrol, the expression gradually reduced and high doses of Res protein expression level reducing is most obvious (Fig. 5C–E).

### Resveratrol enhanced mtROS generation through declining mitochondrial oxdative stress related proteins SIRT3 and MnSOD expression

**Figure 6:**
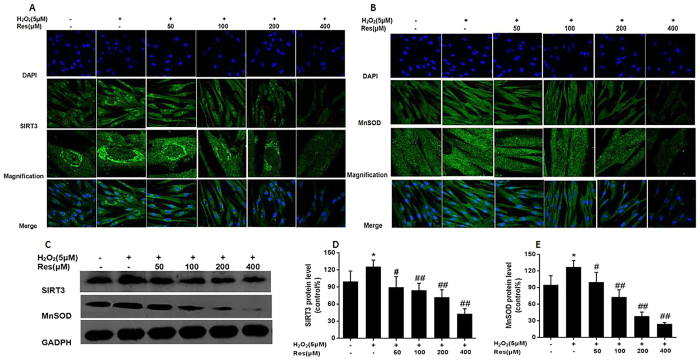
Resveratrol enhanced mtROS generation through declining mitochondrial oxidative stress related proteins SIRT3 and MnSOD expression. Cells were treated with different concentrations (0, 5, 10, 20, 40, 80 and 100 μM) H_2_O_2_ for 12 h and cells were pretreated for 24 h with various concentrations of Res (0, 50,100,200,300 and 400 μM) before treatment with 5 μMH_2_O_2_ incubation 12 h, respectively. (**A,B**) Representative images show mitochondrial SIRT3 and MnSOD proteins localization in FLSs by immunofluorescence assay. Green fluorescence indicates SIRT3 and MnSOD. Blue fluorescence indicates nucleistained with DAPI. (**C**) After the indicated treatments, the protein was harvested to detect SIRT3 and MnSOD levels by western blot analysis. (**D,E**) SIRT3/GADPH and MnSOD/GAPDH. Values are the means ± SD of at least three independent experiments. *P < 0.05 versus control; ^#^P < 0.05 versus 5 μMH_2_O_2_ group.

Immunofluorescence images indicated that the SIRT3 protein was located in the cytoplasm and nucleus. MnSOD protein merely enriched in the cytoplasm. However, the 5 μM H_2_O_2_ group SIRT3 protein mainly enriched in nucleus surrounding, especially the nucleus surrounding fluorescence is very strong and with increasing concentration of Res, SIRT3 and MnSOD fluorescence intensity gradually reduced (Fig. 6A,B). Western blotting analysis, compared with control group 5 μMH_2_O_2_ can increase the expression of SIRT3 and MnSOD protein. With increasing concentration of Res, the expression gradually reduced and high doses of Res protein expression level reducing is most obvious (Fig. 6C–E).

## Discussion

The main features of RA patients rheumatoid arthritis showed the formation of pannus invasion and destruction of articular structure in local thickening of the synovium. Pannus is mainly composed of permeate combination of synovial fibroblasts and a large number of lymphocytes and macrophages^33^,^34^. Human FLSs plays an important role in the pathogenesis of RA. TNF-α and IL-1β are extremely important in the induction of arthritis, can trigger the production of matrix metalloproteinases (MMPs), and ultimately damage the synovial and articular cartilage^35^, In addition, macrophages and FLSs can produce proinflammatory cytokines such as TNF-α, IL-1β, IL-6, IL-8. Excessive production of pro-inflammatory cytokines stimulates neutrophils and activated macrophages the secreting ROS which making them as the medium of joint damage^36^,^37^. So the accumulation of ROS leads to the arthritis which is the main reason. Under the condition of oxidative stress, ROS was produced in excess leading to lipid peroxidation, protein oxidation and DNA fragmentation^38^. MDA, a marker for lipid peroxidation, is frequently used as an indicator for measurement of cellular membrane damage^39^. Superoxide anion is regulated by enzymes such as superoxide dismutase (SOD) and peroxidases, as well as endogenous supplies of antioxidants such as glutathione (GSH)^40^.

Resveratrol is a phenolic compound, which is widely found in various fruits and vegetables, possess antioxidant, anti-aging, regulation of lipid metabolism, anti-cancer properties^18^. Similarly, *in vivo* experiments, it was found that the content of MDA in the model group was significantly higher than the normal group, and the 5mg/kg, 15 mg/kg, 45 mg/kg resveratrol group and 200 mg/kg NAC group were significantly decreased in AA rats serum. The total SOD activity, total antioxidant capacity and the ratio of GSH-PX and GSH reductase in the 15 mg/kg resveratrol group were significantly lower than the normal control group, after successively intragastric administration of 5 mg/kg, 15 mg/kg, 45 mg/kg resveratrol and 200 mg/kg NAC, those were significantly higher. HE staining showed that 15 mg/kg, 45 mg/kg resveratrol and 200 mg/kg NAC can significantly suppressed the AA rat knee joint synovium hyperplasia, relieved the pannus formation and synovial endothelial swelling, also can effectively reduce the subsynovial layer of a large number of lymphocytes and plasma cells infiltration, inhibited synovial invasion of cartilage and subchondral substrate. Especially 45 mg/kg resveratrol improved more obviously. Thus, we further confirmed that AA rats had oxidative stress. This oxidative stress can promote the proliferation of synovial cells and the thickening of the synovial layer. However, the resveratrol could inhibit proliferation of the synovial layer in AA rats.

In recent years, more and more evidences show that resveratrol can promote the apoptosis of cancer cells^41^. High concentrations of resveratrol can induce many kinds of tumor cells apoptosis. FLSs has the characteristics of tumor like properties. Therefore, we also show that the apoptotic effect of 50–200 μM resveratrol on FLSs were not obviously, but the 200–400 μM resveratrol significantly promoted the apoptosis of FLSs, and even 400 μM of resveratrol obviously increased the apoptosis rate of FLSs. In addition, mitochondria can also regulate the apoptosis of tumor cells. In the physiological state, more than 90% of ROS are produced by the mitochondria, and in the process of respiratory chain transfer, the electron from the mitochondrial respiratory chain of the mitochondrial respiratory chain is formed by the oxidation of the complex of the oxidative phosphorylation of the complex I and III is the formation of O_2_▪^− ^42. O_2_▪^−^ mainly through manganese superoxide dismutase (MnSOD) to form H_2_O_2_; H_2_O_2_ was decomposed into H_2_O by the body’s hydrogen peroxide enzymes. There is a good balance between them, once the balance is broken, it will lead to the occurrence of oxidative stress, resulting in a large number of active ingredients. In our experiment, we found excessive H_2_O_2_ to cause early apoptotic FLSs and late apoptotic/necrotic FLSs increasing and we also found that resveratrol can increase mtROS generation and early apoptotic cells and late apoptotic/necrotic cells in H_2_O_2_-treatmnet FLSs. So ROS plays a very important role in cell proliferation and apoptosis. At low concentrations, ROS can be used as a signal molecule to regulate cell proliferation and other functions of which can lead to cell senescence and death at high concentrations of ROS^43^.

Resveratrol interferes into the signal pathway in tumor cells to regulate cell survival and apoptosis, but also regulates the mitochondrial permeability, damage the mitochondrial membrane potential^44^,^45^, the destruction of electronic respiratory chain^46^, and increase the production of mitochondrial superoxide. In our experiment, the mtROS and mitochondrial membrane potential (Δψm) were significantly increased comparing with normal control group. With the increase of the concentration of resveratrol, and the apoptosis rate was higher. Recent studies have found that resveratrol can cause the change of SIRT3 in the SIRTs family, SIRT3 plays a key role in regulating the balance of MnSOD in cells. It is used to change the content of mitochondrial ROS by MnSOD^47^. In our experiments, 5 μMH_2_O_2_ was found to promote the expression of SIRT3 and MnSOD protein, and MnSOD protein expression decreased with the increasing resveratrol concentration, which indicated that the antioxidant capacity of SIRT3 was decreased. This also further suggests that mitochondrial ROS content is increased. This means that MtROS could trigger early apoptotic cells and late apoptotic/necrotic cells in H_2_O_2_-treatmnet FLSs. In addition, ROS plays a very important role in the induction of autophagy, and it is likely that transcription factors are regulated by oxidative regulation of autophagy. Recently, it was found that resveratrol can induce FLSs apoptosis in RA patients with caspase-8 and caspase-9 in the caspase pathway, which activates the caspase-3 pathway. And then, Bcl-2, Bcl-xL or Bax activity can also be changed in the mitochondrial membrane and cytoplasm, and then promote the apoptosis of cells^48^. In addition, studies have found that, under normal conditions, autophagy related proteins Beclin1, AMBRA1 and Bcl-2 or Bcl-XL combine together. When BH3-only was combined with Bcl-2 or Bcl-XL, Beclin1 and AMBRA1, VPS34 and could be formed by the combination of and VPS15, extend the wrapping of mitochondria in LC3-II under the action of mature and lysosomal binding, mitochondrial autophagy triggered^49^. In order to further understand the signal pathway of resveratrol induced apoptosis in FLSs, we studied the expression levels of LC3A/B protein and Beclin1 protein associated with autophagy maker. The results showed that after treatment with 5 μMH_2_O_2_, the expression level of LC3A/B had no significant change and the expression level of Beclin1 increased in FLSs. With different resveratrol concentration, the expression level of LC3A/B and Beclin1 decreased. Therefore, the signaling pathway of FLSs is likely to be caused by the accumulation of MtROS by autophagy pathway and oxidative stress combined action, and excessive MtROS induced apoptosis in FLSs. To sum up, resveratrol plays a very important role in the inhibition of abnormal proliferation of FLSs in AA rats, but the specific detailed mechanism of its induction of apoptosis is not clear, so the results of this study can provide a theoretical basis for the further study on the mechanism of apoptosis, and it is very important to the pathogenesis and treatment of RA.

## Materials and Methods

### Chemicals and reagents

Resveratrol was purchased from Aladdin (Shanghai, China). NAC (N-acetyl-L-cysteine) and Dimethyl sulfoxid (DMSO) were purchased from Sigma (St. Louis, MO, USA). The MDA assay kit, SOD activity assay kit, antioxidant capacity assay kit, glutathione peroxidase assay kit and glutathione reductase assay kit were obtained from Nanjing Jiancheng Bioengineering Institute (Nangjing, China). Dulbecco’s modified eagle medium (DMEM) and fetal bovine serum (FBS) were obtained from Gibco (St Louis, MO, USA). A cell counting kit (CCK-8) was purchased from Biosharp (Hefei, China). Annexin V-FITC/PI staining kit was purchased from BestBio (Shanghai, China). Anti-MnSOD antibody, anti-Sirt3 antibody, anti-LC3A/B antibody and anti-Beclin1 antibody were purchased from Abcam (Beverly, MA, USA). Alexa Fluor 488-conjugated secondary antibody and 4′,6′-diamidino-2-phenylindole (DPI) were purchased from BEIJING BIOSS (beijing, China). MitoSOX Red mitochondrial superoxide indicator and 5,5′,6,6′-Tetrachloro-1,1′,3,3′-tetraethyl-imidacarbocyanine iodide (JC-1) for live-cell imaging was obtained from life technologies (San Diego, CA, USA).

### Experimental animals and design

Male SD rats weighed 180 ± 20 g were provided from laboratory animal Center of Anhui Medical University in this experiment. Rats were acclimatized in temperature and humidity controlled rooms for one week. Rats can be freely available standard food and tap water. The left hind toe of male SD rats were injected with 150 μl Freund’s complete adjuvant (FCA, Sigma, USA) for 20 days. Control rats were injected with 150 μl physiological saline. Then adjuvant-arthritis rats and control rats were randomly assigned to six groups treated with 5 mg/kg, 15 mg/kg, 45 mg/kg resveratrol and 200 mg/kg N-acetyl-L-cysteine (NAC, Sigma, USA) for 12 days by continuous intragastric administration. These doses have been selected based on previously described^50^,^51^. All animals were sacrificed after above treatment 12 days. Pairs of legs was excised, fixed in 4% paraformaldehyde or stored in −80 °C for later analysis. Blood samples were collected, and then 4 °C for the night. Next day, those samples were centrifuged at 3500 rpm/min for 20 min to obtain supernatant fluid which was stored at −80 °C for further analyses.

### Isolation and culture FLSs

FLSs were isolated from model group rats as described previously^52^. In brief, sterile synovial tissue were cut into 1 mm^3^ size, with twice the volume of 0.2%-II type collagenase (Sigma, USA) which contained 10% fetal bovine serum (FBS) digesting 2 to 2.5 h (percussion one time every 30 min). And then using 0.25% trypsin digest 30 min before ending to digestion. Resuspend the cells with DMEM (Gibco, USA) containing 15% fetal bovine serum (Gibco, USA) in the 25 cm^2^ flask, then incubated in a humidified atmosphere containing 5% CO_2_ at 37 °C. All cells used in experiments for the 3–5 passengers.

### The swelling degree of the paw and arthritis index score

The foot volume were measured before each rat left hind toe volume injected FCA. After injection occurs, every 3 or 4 days right hind foot volume measurement were executed by Toe swelling measuring instrument (ZH-YLS-7C, China). The swelling degree of the paw to calculate: swelling of the feet (ml) = model group of right hind foot volume (ml)- control group of right hind foot volume (ml) and medication after foot swelling (ml) = after administration of right hind foot volume (ml)-before administration of right hind foot volume(ml). The degree of arthritis index was determined using a scoring protocol^53^, where by severity was scored on a scale of 0–4, where 0 = absent, 1 = minimal, 2 = mild, 3 = moderate, and 4 = severe.

### Oxidative injury parameters

Lipid peroxidation was evaluated by means of the TBARS (thiobarbituric acid-reactive substances) assay. Malondialdehyde (MDA) comes form Lipid peroxide degradation products which can be combined with thiobarbituric acid (TBA) to form pink products. MDA content was determined by using the MDA assay kit (Nanjing Jiancheng Bioengineering Institute, China) according to the manufacturer’s instructions. briefly, Serum of rats with various reagents blending were incubated in 95 °C water bath for 40 min and subsequently centrifuged at 3000 rpm for 10 min to obtain supernate which was estimated absorbance by spectrophotometry at 532 nm and the values expressed as nmol (mg protein)^−1^. SOD activity, Antioxidant capacity, glutathione peroxidase and glutathione reductase ratio were determined by SOD activity assay kit, antioxidant capacity assay kit, glutathione peroxidase assay kit, glutathione reductase assay kit (Nanjing Jiancheng Bioengineering Institute, Nangjing, China). Simply, serum of rats blend with reagents for several minutes. Respectively, obtaining supernate was detected absorbance by spectrophotometry at 550 nm, 520 nm, 412 nm, 340 nm and the values expressed as percentage.

### Hematoxylin and eosin staining

After 12 days of continuous intragastric administration, AA rats were sacrificed. Knee-joint was harvested and fixed in 4% paraformaldehyde. Then fixed tissue was dehydrated and embedded in paraffin after 6 months of decalcification of knee joint. Knee-joint was cut into 4 micron size and mounted on glass slides. The section was stained with hematoxylin and eosin (Beyotime, Shanghai, China). Images of the stained tissue were obtained using light microscopy.

### Cell growth inhibition assay

Cell viability was performed using cck-8 assays kit (Biosharp, Hefei, China) according to the manufacturer’s protocols. Briefly, the cells was plated in 96-well plates and grown 24 h. Next day, the cells were treated with various concentration of hydrogen peroxide (H_2_O_2_) for 12 h, or after various doses of resveratrol pretreatment in FLSs for 24 h, selected dosage of H_2_O_2_ incubation 12 h. After above treatment, A volume of 10 μl cck-8 was added to those wells. Cells were cultured for 3 h at 37 °C. Then absorbance of the 96-well plates was determined on enzymelinked immunosorbent assay plate reader at 450 nm.

### Cell apoptosis detection by flow cytometry

The apoptotic rate of FLSs was detected by flow cytometry using Annexin V-FITC/PI staining kit (BestBio, Shanghai, China) according to the manufacturer’s protocol. Briefly, FLSs were seeded and incubated in 6-well plates and treated with above dosage and time.after that cells were digested with pancreatic enzymes, resuspended in 400 μl Annexin V binding buffer at a density of 1 × 10^6^cells/ml and incubated with 5 μl Annexin V-FITC and 10 μl propidium iodide (PI) for 15 min at 4 °C in dark. Finally, cells were analyzed by flow cytometry (FACS Calibur, BD Biosciences).

### MtROS assessment

The levels of mtROS were detected by MitoSOX Red Mitochondrial Superoxide Indicator (life technologies, CA, USA). FLSs were grown on glass coverslips and then harvested in 6-well plates. After treated with above dosage and time, the cells were cultured with MitoSOX Red Mitochondrial Superoxide Indicator at 37 °C for 10 min. After staining, wash the cells in fresh growth medium 3 times. Images were collected by confocal laser scanning microscope (model LEICA.SP5-DMI6000-DIC; Leica Microsystems GmbH).The quantitative analysis of the red fluorescence signal was detected by using the built-in evaluation software (Leica LAS AF Lite, Mannheim, Germany).

### Mitochondrial membrane potential (Δψm) determination

Mitochondrial membrane potential (Δψm) was detected using Mitochondrial membrane potential assay kit with JC-1 (life of technology, CA, USA) according to the manufacturer’s protocol. JC-1 probe was accumulated in the mitochondrial matrix polymer which gave off a strong red fluorescence in normal mitochondrion. However, JC-1 probe exists as a monomer at unhealthy mitochondrion which gave off a strong green fluorescence. The cells were grown on glass coverslips. After treated with above dosage and time, the cells were cultured with 5 μg/ml JC-1 at 37 °C for 20 min. After staining, wash the cells 3 times with PBS. The fluorescence intensity was determined by confocal laser scanning microscope (model LEICA.SP5-DMI6000-DIC; Leica Microsystems GmbH). The quantitative analysis of the red/green fluorescence signal was detected by using the built-in evaluation software (Leica LAS AF Lite, Mannheim, Germany). The Δψm was represented as the ratio of red to green fluorescence intensity.

### Western blotting

Cells were seeded and incubated in 6-well plates and treated with above dosage and time and washed with ice-cold PBS and then suspended in 150 μl of RIPA lysis buffer (Beyotime, Shanghai, China). The protein concentration was determined using the BCA assay kit (Beyotime, Shanghai, China). An equal amount of proteins was added into each lane. Proteins were separated using 10% SDS-polyacrylamide gel electrophoresis (SDS-PAGE) and transferred to nitrocellulose membranes. After the membranes were blocked with 5% skim milk for 2 h, the membranes were incubated with the primary antibody overnight at 4 °C, washed with Tris-buffered saline-Tween solution (TBST) 3 times and incubated with a 1:1000 dilution of horseradish peroxidase (HRP)-labeled goat anti-rabbit IgG (Beyotime, Shanghai, China) for 1 h. Finally, bands were detected using enhanced chemiluminescence reagents (BOSTER, Wuhan, China).

### Immunofluorescence staining

FLSs were grown on glass coverslips and then harvested. The coverslips were fixed with 4% (v/v) paraformaldehyde for 30 min at 37 °C and permeabilized with 0.1%Triton X-100 for 20 min at room temperature. The slides were blocked with Immunol Staining Blocking Buffer (Beyotime, Shanghai, China), incubated with anti-LC3A/B antibody, anti-MnSOD antibody, anti-Sirt3 antibody (1:200 dilution, Abcam, MA, USA), anti-Beclin1 antibody (1:300 dilution, Abcam, MA, USA) overnight at 4 °C. After washing with PBS, the slides were incubated for 40 min at 37 °C with a Alexa Fluor 488-conjugated secondary antibody (1:400 dilution; BEIJING BIOSS, Bengjing, China). Finally, the slides were washed twice with PBS and the nuclei were counterstained with 4′,6′-diamidino-2-phenylindole (DAPI, BEIJING BIOSS, Bengjing, China) for 4 min at room temperature. Images of the stained cells were obtained using confocal laser scanning microscopy (model LEICA.SP5-DMI6000-DIC; Leica Microsystems GmbH) and the fluorescence intensity was expressed as the percentage relative to the control group (set as 100%), respectively.

### Statistical analysis

Using SPSS19.0 statistical software, data were expressed by mean ± SD. Three or more groups was compared with single factor analysis of variance (one-way ANOVA) and *t* test was used in the two groups. Values of p < 0.05 and p < 0.01 were considered statistically significant.

### Ethics statement

This study was approved by the Association of Laboratory Animal Sciences and the Center for Laboratory Animal Sciences at Anhui Medical University (Permit Number: 15-0026). All experiments on animals complied with the Guide for the Care and Use of Laboratory Animals by the National Institutes of Health, including all use, care and operative procedures. And all experimental procedures were followed the guidelines for humane treatment set by the Association of Laboratory Animal Sciences and the Center for Laboratory Animal Sciences at Anhui Medical University.

## Additional Information

**How to cite this article**: Zhang, J. *et al*. Autophagy and mitochondrial dysfunction in adjuvant-arthritis rats treatment with resveratrol. *Sci. Rep.* **6**, 32928; doi: 10.1038/srep32928 (2016).
